# Cochrane corner: universal screening for SARS-CoV-2 infection

**DOI:** 10.11604/pamj.supp.2020.37.1.26881

**Published:** 2020-12-16

**Authors:** Duduzile Ndwandwe, Lindi Mathebula, Olatunji Adetokunboh, Raoul Kamadjeu, Charles Shey Wiysonge

**Affiliations:** 1 Cochrane South Africa, South African Medical Research Council, Cape Town, South Africa,; 2 Communicable Disease Control, Department of Health, Western Cape, South Africa,; 3 Department of Science and Innovation-National Research Foundation (DSI-NRF) Centre of Excellence in Epidemiological Modelling and Analysis, Stellenbosch University, Stellenbosch, South Africa,; 4 Department of Global Health, Faculty of Medicine and Health Sciences, Stellenbosch University, Cape Town, South Africa,; 5 UNICEF Program Division, Public Health Emergencies Team, New York, USA,; 6 School of Public Health and Family Medicine, Faculty of Health Sciences, University of Cape Town, Cape Town, South Africa

**Keywords:** Universal screening, COVID-19, screening test accuracy, SARS-CoV-2

## Abstract

**Introduction:**

coronavirus disease 2019 (COVID-19) is caused by the novel coronavirus, severe acute respiratory syndrome coronavirus-2 (SARS-CoV-2). Most people infected with SARS-CoV-2 have mild disease with non-specific symptoms, although a few becoming critically ill with septic shock and multiple organ failure. There is an unknown proportion of infected individuals who remain asymptomatic and infectious. Universal screening for COVID-19 infections to detect individuals who are infected before they present clinically could therefore be an important measure to contain the spread of the disease. We highlight a Cochrane rapid review which assessed the effectiveness and accuracy of universal screening for COVID-19 infection.

**Methods:**

the authors of the Cochrane review searched multiple electronic databases to identify studies reporting on the effectiveness of universal screening and reporting on screening test accuracy. Eligible participants for the review included people who had not sought care for potential COVID-19 symptoms.

**Results:**

the authors included 22 publications, with none of them conducted in Africa. Two modelling studies reported on the beneficial and negative effects of screening; and 20 studies (cohort and modelling) reported data on the accuracy of screening tests. The included studies had wide variability in the baseline prevalence of COVID-19 infection as well as study settings and methods. All cohort studies compared screening strategies to reverse transcriptase-polymerase chain reaction (RT-PCR) as the gold standard. The rapid review suggests that there is low certainty of evidence that screening at travel hubs may slow the importation of infected cases. Furthermore, the review highlights the uncertainty and variation in the accuracy of screening.

**Conclusion:**

given the low accuracy of the tests included in this review, a high proportion of COVID-19 infected individuals may be missed and go on to infect others. In addition, some healthy individuals may be falsely identified as positive, requiring confirmatory testing and potentially leading to the unnecessary isolation of these individuals.

## Introduction

Coronavirus disease 2019 (COVID-19), caused by a novel coronavirus (SARS-CoV-2), is responsible for an ongoing global pandemic. COVID-19 protocols such as universal screening have included strategies to minimise the spread of the virus to identify any of the symptoms suggestive of having been infected [1-3]. The screening protocols include temperature checks or asking about international travel or contact with COVID-19 patients, or performing rapid diagnostic tests. Screening can also occur over the telephone, online, or in person, in homes, clinics, workplaces, airports or schools. Such a strategy has limitation given that some people may be infected with COVID-19 virus during incubation period; variation in the severity and detectability of symptoms once the disease begins to progress; imperfect performance of screening equipment or personnel; or active evasion of screening by travellers [1,4,5]. To limit the spread of this virus, the identification of infected people become critical as a measure to contain the spread of the disease. Infected individuals can seek appropriate healthcare and stay away from others. Screening accompanied by rapid diagnostic tests which are later confirmed by reverse transcriptase-polymerase chain reaction (RT-PCR) test has been broadly implemented as a strategy to prevent further transmission of COVID-19 [2,6]. In this commentary, we discuss a Cochrane rapid review by Viswanathan and colleagues on the effectiveness of universal screening for COVID-19 infection compared with no screening and to determine the accuracy of universal screening in people who have not presented to clinical care for symptoms of COVID-19.

## Methods

The review assessed the effectiveness of universal screening for SARS-CoV-2 infection compared with no screening, and the accuracy of universal screening in people who have not presented to clinical care for symptoms of COVID-19 [7]. Specifically, the review authors assessed the 1) effectiveness of universal screening, screening among people who have not presented to clinical care for symptoms related to COVID-19, for SARS-CoV-2 infection compared with (a) no screening or (b) screening in selected populations based on occupation, geographic setting, or community characteristics? Outcomes include incident cases, missed cases, successfully detected cases, averted cases, reduced transmission, mortality, false alarm, and false reassurance. 2) The accuracy of universal screening strategies among people who have not presented to clinical care for symptoms related to COVID-19 for SARS-CoV-2 infection? Outcomes include sensitivity, specificity, positive predictive value, negative predictive value, the area under the receiver operating characteristic curve. On the Several databases were searched to identify studies to be included.

Searches were conducted in various databases between 04 April 2020 - 26 May 2020 to identifies trials, observational studies and modelling studies assessing the effectiveness and accuracy of screening in the general population. The databases searched were Ovid MEDLINE and the Centers for Disease Control (CDC) COVID-19 Research Articles Downloadable Database, Embase.com, the Central, and the Cochrane COVID-19 Study Register, LitCOVID, and three model repositories (COVID-Analytics, Models of Infectious Disease Agent Study [MIDAS], and Society for Medical Decision Making). The review authors included randomized and nonrandomized studies and modelling studies to answer the question of screening effectiveness. For accuracy, the review authors included study designs on patients that provided information on test accuracy which also included modelling studies. The languages considered were English and Chinese. Screening of articles was conducted followed by data extraction and the assessment of the quality of the included studies conducted in duplicate. The results were synthesized narratively and in a tabular form. Paired forest plot for sensitivity and specificity with 95% confidence interval reported. Grading of Recommendations Assessment, Development and Evaluation (GRADE) approach was used for assessment of certainty of the evidence the synthesized studies.

## Results

The review included 22 publications with 17 cohort studies reporting on diagnostic accuracy and 5 modelling studies reporting on the effectiveness of screening and screening test accuracy. The studies were conducted in the United States of America, Europe, and Asia. The modelling studies that evaluated the effectiveness of screening strategies which included symptoms screening of air travellers and symptomatic laboratory testing of asymptomatic healthcare workers in emergency departments. To assess the effectiveness of screening, one study suggested that symptom screening at travel hubs, such as airports, may slightly slow but not stop the importation of infected cases. The authors assessed risk of bias as minor or with no concerns, and low certainty of evidence, but further downgraded for very serious indirectness. The second modelling study indicated that screening of healthcare workers in emergency departments using laboratory tests may reduce transmission to patients and other healthcare workers. The certainty of evidence was downgraded for high risk of bias (major concerns) and indirectness. No modelling studies reported on harms of screening.

To assess the accuracy of screening, all 17 cohort studies compared an index screening strategy to a reference RT-PCR test. Only one study reported on the accuracy of single point-in-time screening and varied widely in the prevalence of COVID-19, settings, and method of measurement. The overall risk of bias was unclear in 16 out of 17 studies, mainly due to limited information on the index test and reference standard. One study was rated at high risk of bias due to the inclusion of two separate populations with likely different prevalence. For several screening strategies, the estimates of sensitivity came from small samples. All screening strategies in 17 studies with 17,574 people incorrectly identified infected people as healthy; healthy people as infected. Thirteen studies which included 16,762 people incorrectly identified infected people as healthy and healthy people as infected. A similar trend observed in 6 studies with 14,741 people where temperature measurements were taken and asking about international travel, exposure to known infected people and exposure to known or suspected infected people. Also asking about symptoms plus temperature measurement observed in 2 studies with 779 people incorrectly identified infected people as healthy and healthy people as infected. Three studies modelling entry and exit screening in airports indicated 70% of infected travellers being missed while another detected 90% of infections but used an unrealistic scenario. The third study used very unreliable methods which the review authors could not use evidence from this study. The review authors highlight that there are limitations in the confidence of these findings because most studies did not describe their screening methods clearly, some found very few cases of infections and the types of participants and settings varied greatly, making it difficult to judge whether the results apply broadly.

## Discussion

The ability to perform effective screening for COVID-19 infection at the earliest presentation is a critically important first step to curbing the spread of the virus in any setting [8]. COVID-19 has presented challenges with screening infected individuals as many may not present symptoms at the time of screening [1,5,9]. Screening at entry points such as airports may only yield 9% of accurate case detection since many with mild symptoms, asymptomatic, concealed symptoms and pre-symptomatic are likely to be undetected during the screening process [9]. World Health Organization recommends screening for infectious diseases in a two-step process, namely primary and secondary screening, most notable with the Ebola crisis [9]. Primary screening can be performed by trained personnel who may or may not be a medical professional and this includes temperature and symptoms screening [9]. The secondary screening performed by medical professional which include an in-depth interview, laboratory test and secondary temperature screening [9].

African countries have challenges with implementing many of the recommended strategies because of weak public health infrastructures while having the greatest infectious disease burdens beside COVID-19 [10]. It is therefore paramount for African countries to have effective screening strategies for the rapidly spreading COVID-19 to alleviate the burden on healthcare systems. Results from this review have shown very low certainty of evidence of screening methods with most of the studies showing low sensitivity and low specificity for the screening strategies implemented in different studies [7]. Even when strategies were combined, there was still low certainty of evidence that some of these strategies could be effective in many settings [7]. Most countries within the African region have implemented their screening strategies [10] and according to the review authors, the screening strategies are only likely to slow down transmission slightly as few infections could be potentially detected through most of these methods while missing approximately 70% of infections using some of the screening strategies [7]. The authors agree that there is a need for more research on rapid laboratory test combined with screening and for better reference standards to effectively to curb the spread of COVID-19 [7]. Research on cost-effective screening strategies is also needed in African countries given the already strained public health systems.

## Conclusion

The findings from this review suggest that screening at travel hubs may slightly slow the importation of infected cases. However, the uncertainty and variation in the accuracy of screening strategies suggest that a high proportion of infected individuals may be missed and go on to infect others, and some healthy individuals may be falsely identified as positive, requiring confirmatory testing and potentially leading to the unnecessary isolation of these individuals. This becomes critical as many African countries open their borders to resuming economic activity. While the African continent had a relatively low number of cases compared to high-income countries, screening at borders remains the only tool available to ensure that there is no importation of new cases to Africa. As thus, studies are needed to evaluate the utility of rapid laboratory tests, combined screening, and repeated screening. The accuracy of RT-PCR is needed for further research to be used as a reference standard. Given the poor sensitivity of existing approaches, the findings of this review point to the greater emphasis on other ways that may prevent transmissions such as face coverings, physical distancing, quarantine, and adequate personal protective equipment for frontline workers.

### What is known about this topic

- Universal screening for COVID-19 is used as a strategy to assess potential individuals who may need to be isolated and seek medical care;
- The screening method uses a questionnaire accompanied by a temperature check to assess if a person may have been infected with COVID-19;
- Asymptomatic individuals do not present with symptoms which make the screening likely to miss infected individuals.

### What this study adds

- The current screening approach is likely to miss the asymptomatic individual infected with COVID-19;
- As many countries open their borders, this study emphases the need to incorporate multiple screening approaches which incorporate point of care tests for everyone in their screening protocols to ensure that there is a rapid response in the management of cases.
